# Structural and *In Vivo* Studies on Trehalose-6-Phosphate Synthase from Pathogenic Fungi Provide Insights into Its Catalytic Mechanism, Biological Necessity, and Potential for Novel Antifungal Drug Design

**DOI:** 10.1128/mBio.00643-17

**Published:** 2017-07-25

**Authors:** Yi Miao, Jennifer L. Tenor, Dena L. Toffaletti, Stacey A. Maskarinec, Jiuyu Liu, Richard E. Lee, John R. Perfect, Richard G. Brennan

**Affiliations:** a Department of Biochemistry, Duke University School of Medicine, Durham, North Carolina, USA; b Division of Infectious Diseases, Department of Medicine, Duke University School of Medicine, Durham, North Carolina, USA; c Department of Molecular Genetics and Microbiology, Duke University School of Medicine, Durham, North Carolina, USA; d Department of Chemical Biology and Therapeutics, St. Jude Children’s Research Hospital, Memphis, Tennessee, USA; Columbia University

**Keywords:** *Aspergillus fumigatus*, *Candida albicans*, Tps1, fungal pathogens, structural biology, trehalose, trehalose-6-phosphate synthase

## Abstract

The disaccharide trehalose is critical to the survival of pathogenic fungi in their human host. Trehalose-6-phosphate synthase (Tps1) catalyzes the first step of trehalose biosynthesis in fungi. Here, we report the first structures of eukaryotic Tps1s in complex with substrates or substrate analogues. The overall structures of Tps1 from *Candida albicans* and *Aspergillus fumigatus* are essentially identical and reveal N- and C-terminal Rossmann fold domains that form the glucose-6-phosphate and UDP-glucose substrate binding sites, respectively. These Tps1 structures with substrates or substrate analogues reveal key residues involved in recognition and catalysis. Disruption of these key residues severely impaired Tps1 enzymatic activity. Subsequent cellular analyses also highlight the enzymatic function of Tps1 in thermotolerance, yeast-hypha transition, and biofilm development. These results suggest that Tps1 enzymatic functionality is essential for the fungal stress response and virulence. Furthermore, structures of Tps1 in complex with the nonhydrolyzable inhibitor, validoxylamine A, visualize the transition state and support an internal return-like catalytic mechanism that is generalizable to other GT-B-fold retaining glycosyltransferases. Collectively, our results depict key Tps1-substrate interactions, unveil the enzymatic mechanism of these fungal proteins, and pave the way for high-throughput inhibitor screening buttressed and guided by the current structures and those of high-affinity ligand-Tps1 complexes.

## IMPORTANCE

Invasive fungal diseases have emerged as major threats, resulting in more than 1.5 million deaths annually worldwide. This epidemic has been further complicated by increasing resistance to all major classes of antifungal drugs in the clinic. Trehalose biosynthesis is essential for the fungal stress response and virulence. Critically, this biosynthetic pathway is absent in mammals, and thus, the two enzymes that carry out trehalose biosynthesis, namely, trehalose-6-phosphate synthase (Tps1) and trehalose-6-phosphate phosphatase (Tps2), are prominent targets for antifungal intervention. Here, we report the first eukaryotic Tps1 structures from the pathogenic fungi *Candida albicans* and *Aspergillus fumigatus* in complex with substrates, substrate analogues, and inhibitors. These structures reveal key protein-substrate interactions, providing atomic-level scaffolds for structure-guided drug design of novel antifungals that target Tps1.

## INTRODUCTION

Invasive fungal diseases (IFDs) have increased tremendously in immunocompromised patients over recent decades. Two million cases of IFDs are reported annually, with an overall mortality rate higher than 50% (1). Pathogenic fungal species, such as members of the genera *Cryptococcus*, *Candida*, and *Aspergillus*, cause significant burdens to the health care system (2–4). Even in developed countries, the mortality rates of IFDs are as high as 20% to 40% (5). These high mortality rates are attributed mainly to the limited classes of antifungal drugs, emerging drug-resistant strains, and the detrimental side effects of these chemotherapeutics. Further complicating this problem is the close resemblance of key pathways of fungi and mammals to those of bacteria or viruses, inevitably resulting in the major difficulty of identifying novel, nonidentical targets, thereby delaying antifungal drug development. Regardless, fungi do have potential targets that are not present in humans, and computer-aided antifungal target selection ranks the trehalose biosynthetic pathway as a top candidate for novel inhibitor and drug design (6). The absence of this pathway in mammalian cells would likely significantly abrogate drug toxicity (5, 7).

**FIG 1:**
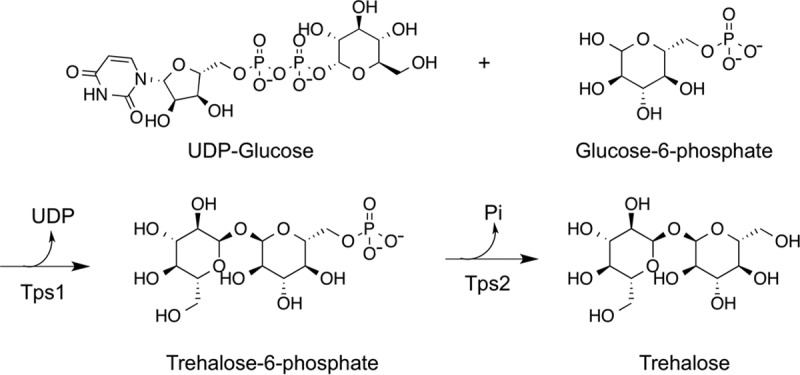
Trehalose biosynthesis pathway in fungi.

Trehalose is a nonreducing disaccharide with two glucose units linked by an α,α-1,1-glycosidic linkage. Fungal cells synthesize trehalose to protect proteins and membranes from external and internal stresses, such as dehydration, heat shock, and oxidation (8–12) A single two-step trehalose biosynthetic pathway has been identified in pathogenic fungi (Fig. 1). The first step, which is catalyzed by trehalose-6-phosphate synthase (Tps1), transfers glucose from UDP-glucose (UDPG) to glucose-6-phosphate (G6P) to form trehalose-6-phosphate (T6P). The second step, which is carried out by the trehalose-6-phosphate phosphatase (Tps2), is the dephosphorylation of T6P to produce trehalose. Disruption of either the TPS1 or TPS2 gene diminishes the ability of *Candida albicans* to adapt or overcome cellular stresses like growth at high temperatures and oxidative stress (13–15). Similar phenotypes are observed in other pathogenic fungi, including *Cryptococcus* (16, 17) and *Aspergillus* (18, 19). Critical to novel drug development against this pathway is that trehalose is synthesized only in bacteria, fungi, lower plants, and invertebrates (20). Collectively, disruption of this pathway has detrimental effects on the survival of these fungi in the host (7).

As a crucial foundation for the rational design of novel inhibitors against these biologically important enzymes in the fungal trehalose biosynthetic pathway, biochemical and structural characterizations of Tps1 and Tps2 are required. However, to date, only the structures of the Tps1 homologue from *E. coli*, OtsA (21–24), and fungal Tps2 (25) have been determined, while no structural information is available for Tps1 from eukaryotes. Here, we describe the first eukaryotic Tps1 structures from the pathogenic fungal species *C. albicans* and *Aspergillus fumigatus* in complex with substrates or substrate analogues and a transition-state analogue. These structures, coupled with in-depth enzyme activity characterization and genetic and cellular studies, have identified key residues for substrate binding, shed light on the enzymatic mechanism, and provided structural scaffolds for inhibitor design and selection.

## RESULTS AND DISCUSSION

### Structure of *C. albicans* Tps1.

**FIG 2:**
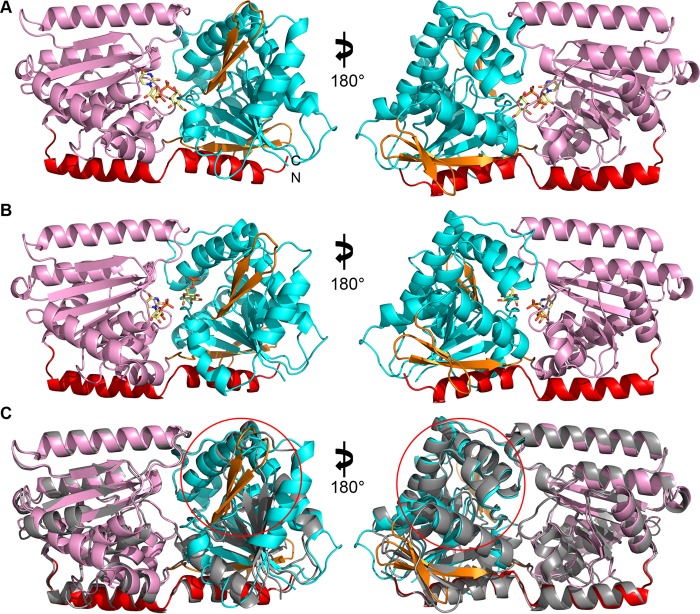
Structures of *C. albicans* Tps1 reveal similarity with the *E. coli* OtsA structure. (A) Cartoon diagram of the *C. albicans* Tps1-UDPG complex. The N-terminal domain is colored cyan, and the C-terminal domain is colored pink. The C-terminal helix interacting with both domains is colored red. The extra antiparallel β-sheet (β2-β3) and the parallel β-sheet (β9-β11) that link the N-terminal and C-terminal domains are colored orange. UDPG is shown as atom-colored sticks with carbon atoms colored yellow, nitrogen atoms colored blue, oxygen atoms colored red and phosphorus atoms orange. This atom-coloring scheme is used for all structural figures throughout the paper. (B) Cartoon diagram of *C. albicans* Tps1-(UDP+G6P) complex. UDP and G6P are shown as atom-colored yellow sticks binding to C-terminal and N-terminal Rossmann fold domains, respectively. (C) Cartoon diagram of the superposition of *C. albicans* Tps1 and *E. coli* OtsA reveals high similarity but some differences. Tps1 is colored as described for panel A, and OtsA is colored grey. Differences between the N termini of these proteins are circled in red. The parallel β-sheet linking the two domains and found in both proteins is also circled in red in the right panel.

The structure of *C. albicans* Tps1 bound to UDPG was determined to 1.90-Å resolution by molecular replacement, using *Escherichia coli* OtsA (PDB code 1UQU) as the search model. The structure of Tps1 in complex with UDP and G6P was determined by molecular replacement using the Tps1-UDPG structure as the search model. Both structures take the hexagonal space group P6_4_ with two subunits in the asymmetric unit. Selected data collection and refinement statistics are listed in Table S1 in the supplemental material. *C. albicans* Tps1 adopts the typical GT-B fold of the retaining glycosyltransferase family, which is characterized by two modified Rossmann fold domains (26). The N-terminal Rossmann fold domain contains a six-stranded parallel β-sheet core that is flanked by eight α-helices (Fig. 2A and B; Fig. S1). Different from a typical Rossmann fold domain, β1 links to an antiparallel β-sheet, composed of strands β2 and β3, instead of an α-helix. In addition, the antiparallel β-sheet, consisting of β9 to β11, links the N-terminal Rossmann fold domain to the C-terminal domain. The C-terminal domain adopts a β/α/β fold with a core of six parallel β strands flanked by eight α-helices. An α-helix at the end of the C-terminal domain extends back into the N-terminal domain, thus interacting with structural elements of both domains. A kink around residue Y457 disrupts the integrity of this helix, which is characteristic of GT-B fold glycosyltransferases. Electron density for UDPG and G6P is detected in the C-terminal and the N-terminal domain, respectively, whereby both binding pockets are close to the subdomain interfaces and consistent with the substrate binding observed in OtsA-ligand complex structures. Superposition of the Tps1-UDPG and Tps1-(UDP+G6P) complex structures reveals no detectable conformational change, indicating that UDPG and UDP induce Tps1 to adopt a closed conformation. Interestingly, no substrate-free (apo) Tps1 protein from any species has been crystallized, suggesting that this form of the protein is flexible. As anticipated, a DALI search (27) of the Tps1-UDPG structure reveals that its highest structural homology is to OtsA from *E. coli*, with a root mean square deviation (RMSD) of 1.8 Å for 452 corresponding Cα atoms. Furthermore, the structural superposition using the DALI pairwise comparison server reveals a highly conserved active site (28). The most dramatic structural differences lie in the N-terminal domain of Tps1, in which β strands β2 and β3 form a β-hairpin in the fungal enzyme that is not present in OtsA (Fig. 2C).

10.1128/mBio.00643-17.1 FIG S1  Structure-based sequence alignment of *C. albicans* Tps1 with Tps1 proteins from other species. Tps1 sequences of *C. albicans* (accession number Q92410), *Cryptococcus neoformans* (accession number Q6IVK9), *A. fumigatus* (accession number Q4WHW0-Tps1B), and *E. coli* (accession number P31677) are aligned. The sequences are annotated as *Tps1_CA*, *Tps1_CN*, *Tps1_AF*, and *Tps1_EC*, respectively. The secondary structure of *C. albicans* Tps1 is shown above the sequences, whereby α-helices are depicted by arrowheads and β strands by rectangles. Identical residues found in all four Tps1 proteins are contained in blue boxes, while those identical in 3 of 4 proteins are contained in light blue boxes. The secondary structure elements are colored as described in the legend to Fig. 2. Key substrate binding residues are highlighted by red asterisks. The sequences are taken from the UniProt Knowledgebase (UniProtKB). Download FIG S1, DOCX file, 1.2 MB. Copyright © 2017 Miao et al. 2017 Miao et al. This content is distributed under the terms of the Creative Commons Attribution 4.0 International license.

10.1128/mBio.00643-17.6 TABLE S1  Selected *C. albicans* Tsp1 data collection and refinement statistics. Download TABLE S1, DOCX file, 0.1 MB. Copyright © 2017 Miao et al. 2017 Miao et al. This content is distributed under the terms of the Creative Commons Attribution 4.0 International license.

### Substrate binding pocket of *C. albicans* Tps1.

**FIG 3:**
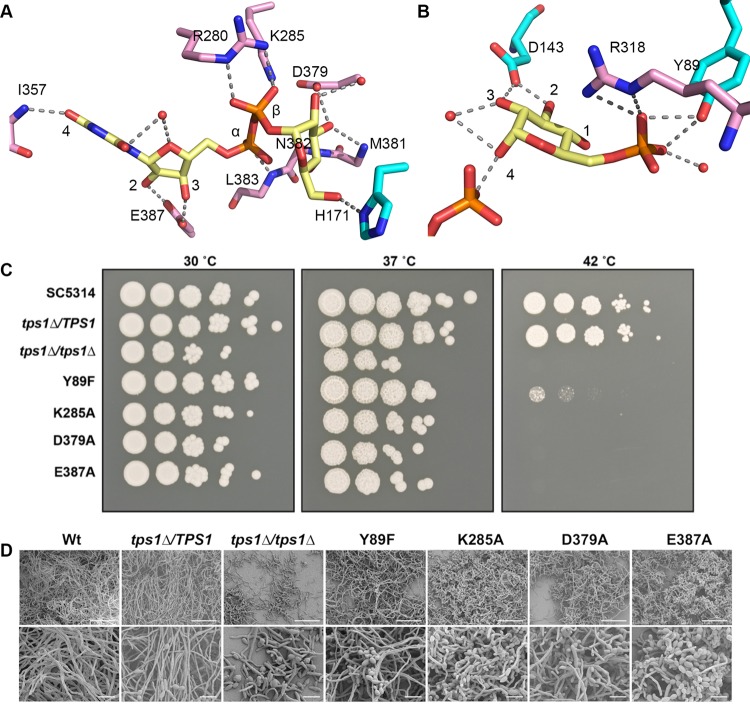
Tps1 residues involved in substrate binding. (A) View of residues and solvent involved in UDPG binding. UDPG is shown as atom-colored yellow sticks. Residues from the N-terminal domain and C-terminal domain of Tps1 are shown as atom-colored sticks as described for Fig. 2 except that the carbon atoms are colored cyan and pink, respectively, for each domain. Hydrogen bonds are shown by dashes. (B) View of residues and solvent involved in G6P binding. G6P is shown as atom-colored yellow sticks. (C) *C. albicans tps1Δ/tps1Δ* and mutants with point mutations are temperature sensitive. (D) Biofilm formation is negatively impacted in mutated TPS1 strains. Biofilm morphology was assessed by scanning electron microscopy at 1,500 µm (top; white scale bar = 50 μm) and 5,000 µm (bottom; white scale bar = 10 μm) for each strain.

Each Rossmann fold domain of Tps1 hosts the binding site of one substrate (Fig. 3A and B). UDPG binds to the C-terminal domain, with the uracil base interacting specifically with Tps1 through a hydrogen bond between its exocyclic O4 atom and the backbone amide group of residue I357 (Fig. 3A). The exocyclic O2 atom is interacting with a water molecule in the active site. Although there are not extensive interactions between uracil and Tps1, interaction between the uracil O4 and Tps1 discriminates UDP from CDP, as the CDP N4 amide group would not be able to form a hydrogen bond with the amide nitrogen of the I357 backbone, which is also a hydrogen bond donor. In addition, the size of the UDPG binding pocket would favor a pyrimidine over the bulkier purines adenine and guanine. Taken together, UDPG is clearly the preferred substrate for *C. albicans* Tps1. Adding to the substrate recognition and binding affinity is the ribose ring of UDP, as the O2 and O3 hydroxyl groups form hydrogen bonds with the side chain of residue E387. With regard to the phosphate moiety of UDP, one hydrogen bond is made between its α phosphate group and the backbone amide group of residue L383. A conserved Arg-Lys pair (residues R280 and K285) interacts with the β phosphate. These interactions are significant for positioning the UDPG in the correct conformation for the activity and subsequent stabilization of the UDP leaving group (Fig. 3A). No monovalent or divalent cation is detected in the electron density, indicating that GT-B fold retaining glycosyltransferases utilize a metal-independent catalytic mechanism.

Somewhat surprisingly, the electron density is poor for the glucose moiety of the UDPG substrate; however, all extracyclic hydroxyl groups are easily detected in electron density maps (Fig. S2A). These hydroxyl groups participate in hydrogen bonds to Tps1, with the O2 hydroxyl hydrogen bonding to a water molecule, the O3 hydroxyl hydrogen interacting with the peptide backbone of residue M381 and the side chain of residue D379, the O4 hydroxyl hydrogen bonding with the peptide backbone of residue N382, and the O6 hydroxyl group hydrogen bonding to the side chain of residue H171. These interactions also highlight the significance of the glucose unit front-side conformation for enzymatic activity. However, without the other substrate, G6P, bound, the glucose moiety of the UDPG is likely more flexible, thereby contributing to the relatively poor electron density observed for this sugar. Finally, the Tps1 structure in complex with UDPG alone suggests Tps1 uses an ordered binding mechanism, with UDPG binding first. No Tps1-G6P complex from any species has been crystallized despite intense efforts.

10.1128/mBio.00643-17.2 FIG S2  Electron density of the UDPG and (UDP+G6P) substrates/product in the structure of the Tps1-UDPG and Tps1-(UDP+G6P) complexes. (A, B) 2F_o_-F_c_ electron density maps of the bound substrates. The electron density is shown as a light blue mesh and contoured at 1.5 σ. Substrates are shown as atom-colored sticks. Download FIG S2, DOCX file, 0.6 MB. Copyright © 2017 Miao et al. 2017 Miao et al. This content is distributed under the terms of the Creative Commons Attribution 4.0 International license.

In order to locate the G6P binding site, the structure of Tps1 in complex with UDP and G6P was solved to 2.37-Å resolution. The most notable interactions between G6P and Tps1 are between the phosphate moiety and side chains of residues Y89 and R318 (Fig. 3B). Another major contributor is residue D143, the side chain of which interacts with the O2 and O3 hydroxyl groups. Compared to the UDP product, G6P shows weaker electron density in the Tps1-(UDP+G6P) structure (Fig. S2B). This weaker density is a likely consequence of substituting UDP for UDPG, hence allowing additional flexibility in the binding pocket. Importantly, the structures of the Tps1-UDPG and Tps1-(UDP+G6P) complexes indicate that the enhanced mobility of the glucose moieties of UDPG and G6P would facilitate substrate positioning to enforce the appropriate stereochemistry and allow efficient catalysis.

To assess further the universality of the substrate binding of Tps1 proteins, the Tps1 sequence from *C. albicans* was aligned with those of *E. coli*, *Cryptococcus neoformans*, and *A. fumigatus* (Fig. S1). Residues involved in substrate binding are conserved among these species except for I357, the side chain of which is involved not in UDP recognition but, rather, its peptide backbone. This conservation of all residues strongly supports the idea that all Tps1 proteins adopt the same binding pocket for both substrates and utilize identical catalytic mechanisms. Intriguingly, the fungal Tps1 homologues are of similar molecular weights, with the exception of the Tps1 enzyme from *C. neoformans*, which has approximately 200 extra residues located throughout the protein. These extra residues might compose additional functional domains of the *C. neoformans* Tps1. However, their structures and any possible function remain enigmatic.

### Significance of key Tps1-interacting residues in catalysis and stress response.

To dissect the significance of the conserved substrate binding residues, four representative mutations were introduced individually into *C. albicans* Tps1. Coupled enzyme assays (Fig. S3) were performed on *C. albicans* Tps1 mutants with the mutations Y89F (the change of Y to F at position 89), K285A, D379A, and E387A. Compared with that of the wild-type protein, no enzymatic activity was detected for these mutants. Thus, the identity of these substrate-binding residues is essential for Tps1 activity. Additionally, these mutations were introduced into *C. albicans* by replacing the wild-type allele in the *tps1Δ/TPS1* strain with the TPS1 gene carrying the designated nucleotide changes conferring the changes in codons. At 37°C, a *tps1Δ/tps1Δ* deletion strain and the strains with K285A, D379A, and E387A substitutions but not the strain with the Y89F substitution showed temperature sensitivity (Fig. 3C). This effect was even more pronounced at 42°C, where the Y89F mutant was also temperature sensitive, indicating that specific Tps1 enzymatic activity is essential for fungal adaptation to elevated temperatures (Fig. 3C). The transition of yeast cells to the hyphal form is critical for *C. albicans* to cause disease (29–31). Therefore, we examined the ability of these strains carrying point mutations in *C. albicans* Tps1 to form hyphae in liquid medium. Tps1 mutants with the K285A, D379A, and E387A mutations were less able to form hyphae in the presence of serum at 37°C after 4 h of incubation (Fig. S4). The Y89F mutant was able to form hyphae, but the presence of yeast cells suggested that this mutant was slightly impaired in hypha formation. Candida forms biofilms, and these biofilms are more resilient to antifungal drug treatment. Using scanning electron microscopy, we evaluated the *C. albicans* Tps1 mutants for their ability to form biofilms and observed that all *C. albicans* Tps1 mutants were impaired in biofilm development (Fig. 3D). The most severe phenotype was observed for the *tps1Δ/tps1Δ* mutant. This strain was unable to form hyphae, as noted by the absence of hyphae after 22 h of growth at 37°C and the presence of yeast cells and pseudohyphae. The heterozygous strain, *tps1Δ/TPS1*, was able to form hyphae. The *C. albicans* Tps1 point mutants with the Y89F, K285A, D379A, and E387A mutations developed abnormal biofilms, as observed by the presence of yeast cells and pseudohyphae on the exposed contact lens surface and limited presence of hyphae. Similar to the results of the liquid serum hypha assay, the Y89F mutant had the weakest phenotype, as yeast cells and hyphae were observed.

10.1128/mBio.00643-17.3 FIG S3  Relative catalytic activities of *C. albicans* WT Tps1 and selected mutants. Error bars represent standard errors of the results of three independent measurements. Download FIG S3, DOCX file, 0.1 MB. Copyright © 2017 Miao et al. 2017 Miao et al. This content is distributed under the terms of the Creative Commons Attribution 4.0 International license.

10.1128/mBio.00643-17.4 FIG S4  Effect of *C. albicans* Tps1 point mutations on formation of hyphae in the presence of serum. A *tps1Δ/tps1Δ* mutant is defective in its ability to form hyphae, as previously reported. Strains carrying point mutations K285A, D379A, and E387A also failed to form hyphae. Yeast cells are present for Y89F, suggesting that this mutation does impact hyphal formation but to a lesser degree than the other single point mutations. Overnight cultures were washed, transferred to YPD containing 10% fetal bovine serum, and incubated at 37°C for 4 h with shaking. The scale bars represent 20 µm. Download FIG S4, DOCX file, 0.2 MB. Copyright © 2017 Miao et al. 2017 Miao et al. This content is distributed under the terms of the Creative Commons Attribution 4.0 International license.

Altogether, these results highlight the significance of these substrate-interacting residues in the biosynthetic activity of Tps1 and suggest that the enzymatic activity of Tps1 is essential for *C. albicans* thermotolerance and the morphological transitions necessary for invasive disease production. These results further designate Tps1 as a novel antifungal target. Our structures of the first eukaryotic Tps1 proteins from *C. albicans* should serve as excellent scaffolds for structure-based inhibitor design.

### Elucidation of catalytic mechanism of Tps1.

**FIG 4:**
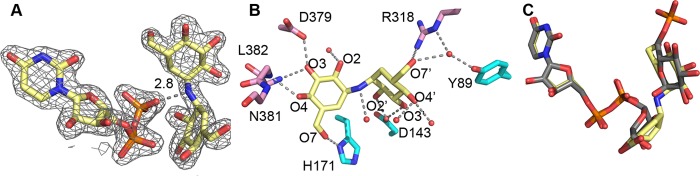
Structure of the *C. albicans* Tps1-(UDP+VDM) complex. (A) A 2F_o_-F_c_ electron density map of the Tps1-bound UDP and VDM, shown as light blue mesh and contoured at 1.5 σ. VDM is shown as atom-colored sticks as described for Fig.1. (B) View of interactions involved in VDM binding. Residues of Tps1 from the N-terminal domain and C-terminal domain are shown as sticks and colored as described for Fig. 3. The oxygen atoms are labeled with primed numbers describing the oxygens of the other six-membered hexose moiety. Water molecules are shown as red spheres. Hydrogen bonds are shown by dashes. (C) Superposition of UDPG from the Tps1-UDPG complex structure and G6P from the structure of the Tps1-(UDP+G6P) complex onto UDP and VDM from the Tps1-(UDP+VDM) complex structure. UDP and VDM are shown as atom-colored sticks. UDPG and G6P are shown as atom-colored sticks with carbon atoms colored grey.

Tps1 is a member of the GT-B fold retaining glycosyltransferases (26). Although well studied, the catalytic mechanism of Tps1 remains somewhat unclear. The *C. albicans* Tps1 structures bound to either UDPG or UDP and G6P show poor electron density for the glucose moieties of UDPG and G6P (Fig. S2). The lack of highly accurate structural information that would allow a better description of the stereochemistry of UDPG and G6P binding in the active site hinders our fullest understanding of the enzymatic mechanism of fungal Tps1 proteins. In an attempt to overcome this obstacle, Tps1 was crystallized with a transition-state inhibitor, validoxylamine A (VDM) and UDP, which together form a mimetic of the Tps1 transition state (32). We note that although VDM is a known trehalase inhibitor, the term “inhibitor” is used here because validoxylamine A is able to diminish Tps1 activity in the presence of UDP (21). The structure of the complex was solved to 1.80-Å resolution by molecular replacement, utilizing the *C. albicans* Tps1-UDPG structure as the search model. VDM is a nonhydrolyzable inhibitor with a structural scaffold that resembles that of trehalose. Unambiguous electron density is observed for the VDM molecule (Fig. 4A). Each of the VDM hydroxyl groups engages in extensive interactions with Tps1 and solvent (Fig. 4B). Not surprisingly, the VDM hydroxyl groups share identical interactions with Tps1, as do its natural substrates. Interestingly, the absence of a phosphate moiety in VDM results in a water-mediated interaction between its 7′ hydroxyl group and the side chains of residues Y89 and R318. Furthermore, superposition of our three *C. albicans* Tps1 structures, Tps1-UDPG, Tps1-(UDP+G6P), and Tps1-(UDP+VDM), clearly reveals retention of the anomeric configuration upon catalysis (Fig. 4C).

Two different enzymatic mechanisms, the double displacement mechanism and the internal return mechanism, have been proposed for GT-B fold retaining glycosyltransferase (26). The double displacement mechanism requires an active-site residue to serve as a nucleophile. However, none of the Tps1 structures shows any nucleophile positioned in proximity to effect bond cleavage. Therefore, an internal return mechanism is strongly favored and is supported by the Tps1-(UDP+VDM) structure, in which the β phosphate of UDP is located 2.8 Å from the pseudoglycosidic bond of VDM (Fig. 4A). This interaction and orientation favor a front-side, internal return-like (S_N_-i) mechanism, with UDPG deprotonating G6P. This mechanism has been proposed and experimentally characterized for OtsA, the *E. coli* homologue of Tps1 (21, 32).

### Structures of *A. fumigatus* Tps1A and Tps1B.

In order to test our hypothesis that the Tps1 proteins from other pathogenic fungi employ the same enzymatic mechanism as that observed for the *C. albicans* Tps1 and *E. coli* OtsA, the Tps1 enzymes from *C. neoformans* and *A. fumigatus* were purified and crystallized with substrate analogues. Despite multiple efforts, *C. neoformans* Tps1 failed to yield diffraction-quality crystals. In contrast, Tps1 structures from *A. fumigatus* have been obtained. Different from *Cryptococcus* and *Candida*, *A. fumigatus* contains two trehalose-6-phosphate synthase genes (annotated here as Tps1A and Tps1B). Both structures were determined in complex with UDP and VDM to obtain accurate information on the substrate stereochemistry and active-site residue–substrate interactions.

The structure of the *A. fumigatus* Tps1A-(UDP+VDM) complex was determined to 2.80-Å resolution by molecular replacement using the structure of *C. albicans* Tps1-UDPG as the search model. Two molecules were present in the asymmetric unit, and electron density was not visible for residues 1 to 11, 35 to 39, 56 to 63, 68 to 70, and 480 to 515 of chain B. The presence of a disordered C terminus (residues 480 to 515) is consistent with secondary structure prediction by PSIPRED (33). The structure of the Tps1B-(UDP+VDM) complex was determined to 2.46-Å resolution with only 20 residues (1 to 11, 29 and 30, 38 to 43, and 479 of chain A) missing from the structure. Selected crystallographic data and refinement statistics are listed in Table S2.

10.1128/mBio.00643-17.7 TABLE S2  Selected *A. fumigatus* Tps1A and Tps1B data collection and refinement statistics. Download TABLE S2, DOCX file, 0.1 MB. Copyright © 2017 Miao et al. 2017 Miao et al. This content is distributed under the terms of the Creative Commons Attribution 4.0 International license.

**FIG 5:**
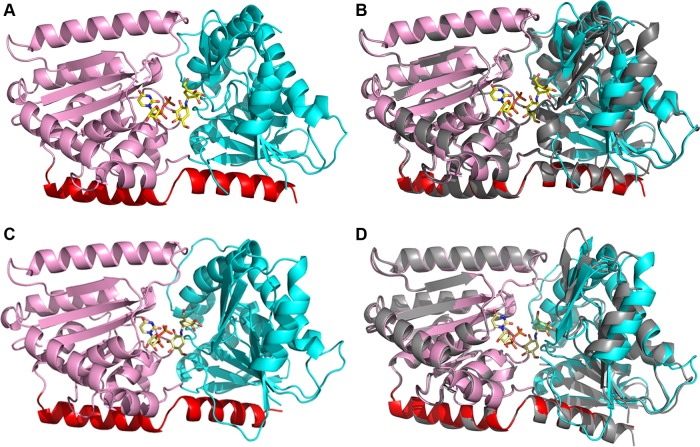
Structure of *A. fumigatus* Tps1A and Tps1B bound to UDP and VDM. (A) Cartoon of the structure of the *A. fumigatus* Tps1A-(UDP+VDM) complex. The N-terminal and C-terminal Rossmann fold domains are depicted as ribbons and colored cyan and pink, respectively, while the C-terminal helix that interacts with both domains is colored red. UDP and VDM are shown as atom-colored sticks. (B) Superposition of the structures of the *A. fumigatus* Tps1A-(UDP+VDM) complex and *C. albicans* Tps1-(UDP+VDM) complex. The former is colored as described for panel A, and the latter is shown as a grey-colored cartoon. (C) Cartoon of the structure of the *A. fumigatus* Tps1B-(UDP+VDM) complex. The N-terminal and C-terminal Rossmann fold domains are depicted as ribbons and colored cyan and pink, respectively, while the C-terminal helix that interacts with both domains is colored red. UDP and VDM are shown as atom-colored sticks. (D) Superposition of the *A. fumigatus* Tps1B and Tps1A-(UDP+VDM) complexes with the latter shown as a grey-colored cartoon.

The overall structures of both Tps1A and Tps1B contain N- and C-terminal Rossmann fold domains and the C-terminal helix that crosses and interacts with each Rossmann fold domain (Fig. 5). This overall structure fold is similar to other GT-B fold retaining glycosyltransferases (26). Structural superpositions of *A. fumigatus* Tps1A with Tps1B reveal a root mean square deviation (RMSD) of 0.52 Å for 452 corresponding Cα atoms (Fig. 5B). Superpositions of *A. fumigatus* Tps1A and Tps1B and *C. albicans* Tps1 reveal RMSDs of 0.48 and 0.41 Å for 452 and 459 corresponding Cα atoms, respectively (Fig. 5D). The most notable difference between *A. fumigatus* Tps1A and Tps1B is the presence of 34 additional residues at the C terminus of Tps1A, none of which are visible in the structure (Fig. S5). The C-terminal tail of Tps1A is replete with serine and threonine residues, suggesting the potential for posttranslational modification of one or more of these sites. The presence of this additional C-terminal tail also suggests different functions of Tps1A and Tps1B despite the fact that both need to be knocked out to affect the stress response of *A. fumigatus in vivo* (18). Hence, the function of this tail remains obscure and further research is required to understand its role, if any, in the stress response of *A. fumigatus*.

10.1128/mBio.00643-17.5 FIG S5  Sequence alignment of *A. fumigatus* Tps1A and Tps1B. Primary sequences of *A. fumigatus* Tps1B and Tps1A are aligned. Identical sequences are shaded blue. The secondary structure of Tps1B is shown above the alignment. Arrows represent α-helices, and rectangles β strands. The secondary structure elements are colored as described in the legend to Fig. 5. Note the C-terminal extension of Tps1A. Download FIG S5, DOCX file, 0.8 MB. Copyright © 2017 Miao et al. 2017 Miao et al. This content is distributed under the terms of the Creative Commons Attribution 4.0 International license.

### Substrate recognition of *A. fumigatus* Tps1A and Tps1B.

**FIG 6:**
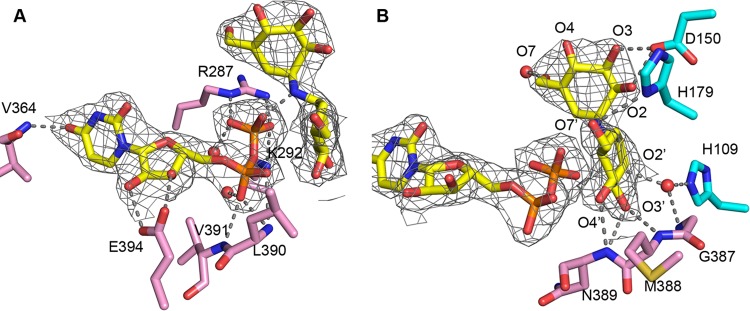
Active-site interactions of the *A. fumigatus* Tps1B-(UDP+VDM) complex. (A, B) two views of the active site interactions. 2F_o_-F_c_ electron density maps of the bound UDP and VDM are shown as grey mesh and contoured at 1 σ. UDP and VDM are shown as atom-colored sticks and solvent as red spheres. Residues originating in the N-terminal Rossmann fold have cyan-colored carbon atoms, and those from the C-terminal Rossmann fold have pink-colored carbon atoms. Protein-substrate and water-substrate interactions are indicated by dashed lines.

To visualize the interactions of *A. fumigatus* Tps1 with substrates, Tps1A and Tps1B were both crystallized with UDP and VDM, which allowed them to be captured in their transition-state conformations (21, 32). The electron density of these ligands is unambiguous in the conserved catalytic pocket of both proteins (Fig. 6). Sequence alignment and structure superposition reveal highly conserved active-site interactions between these two proteins (Fig. S5). To avoid a redundant description of the protein-ligand interactions, we focus on describing the higher-resolution Tps1B interactions with UDP and VDM, in which solvent molecules are better resolved and, hence, provide a more comprehensive and accurate view of the active site (Fig. 6).

The uracil base of UDP makes only one hydrogen bond to Tps1B, from its O4 oxygen to the amide group of residue V364 (Fig. 6A). This contact selects uracil over cytosine. Despite the seeming dearth of base-specifying contacts, the size of the Tps1 substrate pocket favors a smaller pyrimidine over the larger purines, and thus, UDPG is one of the preferred substrates of Tps1A and Tps1B. The positioning of UDP is also dependent on the interactions of its ribosyl 2′ and 3′ hydroxyl groups with the side chain of residue E394. UDP binding is stabilized further by hydrogen bonds between its α phosphate and the backbone amide groups of residues L390 and V391. The conserved K/R pair (K292 and R287) interacts with the β phosphate and would shield the negative charge of the leaving group during catalysis. Interestingly, the interaction, observed in the *C. albicans* Tps1-(UDP+VDM) complex structure, is also seen between the β phosphate oxygen atom and the N-glycosidic bond of VDM. This interaction further supports the idea that Tps1 utilizes an S_N_-i mechanism (32) involving the deprotonation of G6P by UDPG.

As noted above, VDM is a nonreactive molecular mimic of trehalose, in which the O-glycosidic bond joining the two glucose moieties of trehalose is replaced by a nonhydrolyzable N-glycosidic bond. The positions of the hydroxyl groups on its two pseudoglucose moieties are identical to those observed for the glucoses of UDPG and G6P (Fig. 6B). The O2′ interacts with a water molecule, which is locked in the active site by hydrogen bonds from the side chain of residue H109 and the backbone amide group of residue G387. The O3′ and O4′ hydroxyl groups interact with the backbone amide groups of the loop between α12 and β12. This loop is of critical importance for positioning the α phosphate and glucose leaving groups of UDPG. The O7′ hydroxyl group of VDM makes a hydrogen bond to the side chain of residue H179. Perhaps the key contributor to positioning the pseudoglucose moiety of VDM is residue D150, which interacts with both the O2 and O3 hydroxyl groups of the second hexose ring. The O7 hydroxyl interacts with a water molecule, which mimics the phosphate moiety of G6P.

In summary, we report here the structures of the first fungal and, indeed, the first eukaryotic Tps1 proteins in complex with substrates, product, substrate analogues, or post–transition-state analogues. These structures, together with the results of enzymatic activity assays, highlight key residues for Tps1 activity. Subsequent genetic and *in vivo* analyses reveal that Tps1 enzymatic activity is essential for proper fungal stress response in *C. albicans*. However, the trehalose pathway is complex in *Aspergillus fumigatus*, and while a block of Tps2, the trehalose-6-phosphate phosphatase, leads to attenuation of the strain (19), a TPS1 mutant appears to be hypervirulent (18). This is likely due to the impact of the product of Tps1 on how the cell wall of the mutant is presented to host immunity (34). Therefore, inhibitors of *Aspergillus fumigatus* Tps1, which still may have a detrimental impact on mould survival, will need to be examined for their potential negative treatment features that are not seen with *Candida* and *Cryptococcus*. Regardless, the *C. albicans* and *A. fumigatus* Tps1 and *E. coli* OtsA proteins adopt essentially identical structural folds and make conserved active-site–substrate interactions. Furthermore, the structures of the *C. albicans* and *A. fumigatus* Tps1-(UDP+VDM) complexes strongly support an internal return-like mechanism (S_N_-i) for catalysis, which is utilized by *E. coli* OtsA and likely applies to other prokaryotic and eukaryotic GT-B fold retaining glycosyltransferases. Thus, our current results have deepened significantly our understanding of trehalose biosynthesis in pathogenic fungi and its importance in cell viability and have provided the foundation for designing antifungal inhibitors with potentially low or no off-target effects.

## MATERIALS AND METHODS

### Protein purification and crystallization.

The *tps1* gene from *C. albicans* strain SC5314 and *tps1A* and *tps1B* genes from *A. fumigatus* strain Af293 were codon optimized for expression in *E. coli* (GenScript). These codon-optimized genes were cloned into a pET-28a kanamycin-resistant vector via restriction sites NdeI and SacI. These vectors contained an N-terminal hexahistidine affinity tag followed by a thrombin cleavage site. Each vector was transformed into BL21(DE3)pLysS cells (Life Technologies, Inc.) and induced by the addition of 0.5 mM isopropyl-β-d-thiogalactopyranoside (IPTG) (final concentration) at 15°C for 16 h when the optical density at 600 nm (OD_600_) reached 0.6. The Tps1 proteins were purified by Ni^2+^-nitrilotriacetic acid (NTA) affinity column chromatography followed by thrombin cleavage. Each protein was purified further using size exclusion chromatography via a Superdex S200 column (GE Healthcare) in buffer containing 20 mM Tris, pH 8.0, 200 mM NaCl, 5% glycerol, 5 mM MgCl_2_, and 1 mM β-mercaptoethanol (βME). The resulting purified Tps1 enzymes were concentrated using Amicon Ultra concentrators (30,000 molecular weight cutoff [30K MWCO]; Millipore). To prepare singly and doubly mutated Tps1, a standard site-directed mutagenesis protocol was employed (35). For all crystallization trials, *C. albicans* Tps1 was concentrated to 20 mg/ml and cocrystallized with different substrates or substrate analogues or a transition-state mimic, which was added to the crystallization drop to a final concentration of 10 mM. Crystals were grown at 25°C by hanging-drop vapor diffusion methods. Diffraction-quality crystals appeared after 1 week from solutions containing 0.2 M lithium sulfate, 0.1 M Tris, pH 8.5, and 40% polyethylene glycol (PEG) 400.

Purified *A. fumigatus* Tps1A and Tps1B were concentrated using an Amicon Ultra concentrator (30K MWCO; Millipore) to 12 mg/ml. Tps1A and Tps1B were cocrystallized with 10 mM UDP and VDM. Crystals were grown at 25°C by the hanging-drop vapor diffusion method. Diffraction-quality crystals of the Tps1A-(UDP+VDM) complex appeared after 1 week from solutions of 0.1 M sodium malonate, pH 8.0, 0.1 M Tris, pH 8.0, and 30% PEG 1000. Tps1B-(UDP+VDM) crystals appeared after 3 weeks from solutions of 0.2 M lithium sulfate monohydrate, 0.1 M Tris, pH 8.5, and 20% PEG 3350.

### Synthesis of validoxylamine A.

The title compound was synthesized according to the procedure described by Iwasa et al. (36). Validamycin A (500 mg) in water was refluxed with Amberlite IR-120 (H form, 5 ml) overnight. After filtration, the resin was washed with water (50 ml) to remove all d-glucose. Then, 1 M ammonium hydroxide (20 ml) was used to release validoxylamine A from IR-120 resin. After filtration, the solvent was removed to yield validoxylamine A (210 mg, 62.3%) as an off-white solid. ^1^H nuclear magnetic resonance (NMR) (400 MHz, deuterium oxide): δ 6.08 to 5.91 (m, 1H), 4.30 to 4.08 (m, 3H), 3.76 to 3.56 (m, 4H), 3.39 (t, *J* = 5.0 Hz, 1H), 3.31 to 3.24 (m, 2H), 2.03 to 1.83 (m, 2H), 1.31 (td, *J* = 13.6, 12.2, 2.8 Hz, 1H). Mass spectrometry (MS) (electrospray ionization [ESI]): [M+H]^+^ 336.

### X-ray intensity data collection and structure determination and refinement.

X-ray intensity data sets were collected at the Advanced Photon Source (APS) ID-22 line and were indexed and scaled using HKL 2000 (37). The *C. albicans* Tps1 structure with UDPG was determined by molecular replacement using Phaser (38) and with the OtsA structure as the search model (PDB code 1UQU) (23). The other structures were determined by molecular replacement using the *C. albicans* Tps1-UDPG structure as the search model. All structures were manually built in Coot (39) and improved by multiple rounds of refinement in Phenix (40) and rebuilding. Selected data collection and structure refinement statistics are listed in Tables S1 and S2 in the supplemental material.

### Tps1 enzyme activity assay.

The catalytic activity of Tps1 was measured utilizing a continuous enzyme coupled assay as previously reported (21). Briefly, protein was concentrated and the assay carried out in a buffer containing 50 mM HEPES, pH 7.8, 100 mM KCl, 5 mM MgCl_2_, and 2 mM dithiothreitol (DTT). Final concentrations of 3 μM, 1 mM, and 1 mM were utilized for Tps1, UDPG, and G6P, respectively. Activity assays were performed in Greiner 96-well plates, and the decreases in absorbance at 340 nm were recorded using a SpectraMax M5 plate reader (Molecular Devices). The initial enzymatic activity velocities were plotted and compared between WT protein and mutant proteins.

### Strains and growth conditions.

The *C. albicans* strains used in this study are listed in Table S3. *C. albicans* strains were grown in YPD (1% yeast extract, 2% peptone, and 2% dextrose) medium. Cultures were maintained at 30°C unless otherwise specified. The deletion mutant and strains carrying point mutations in *TPS1* were constructed using the Clox system (41). For deletion of *C. albicans TPS1*, the two *TPS1* alleles were deleted sequentially using the Clox system as described in reference 41. The deletion construct was prepared by an In-Fusion cloning method that fused 3 PCR fragments (a 794-bp upstream fragment plus the start codon, the 4,622-bp NAT1-Clox cassette amplified from the Clox-NAT plasmid [41], and an 887-bp downstream fragment plus the stop codon) into pUC19. The TPS1 point mutations were constructed by PCR followed by In-Fusion cloning. Next, the NAT1-Clox cassette was fused downstream from the mutated gene to allow for recombination at the TPS1 locus. Transformation of *C. albicans* was performed by following the protocol in reference 42. Transformants were selected on YPD plates containing 200 µg/ml nourseothricin (NAT; GoldBio), 2.5 mM methionine, and 2.5 mM cysteine. Methionine and cysteine repress the *MET3* promoter that controls the expression of the recombinase, thereby limiting Cre-*loxP*-mediated recombination. To remove the drug-selectable marker, strains were grown overnight in YPD and streaked onto nonselective medium. Each colony was then confirmed by PCR for loss of the drug marker, loss of TPS1, or insertion of TPS1 carrying a point mutation. The TPS1 point mutations were inserted into the *tps1Δ/TPS1* strain to replace the single wild-type allele.

10.1128/mBio.00643-17.8 TABLE S3  Strains used in this study. Download TABLE S3, DOCX file, 0.1 MB. Copyright © 2017 Miao et al. 2017 Miao et al. This content is distributed under the terms of the Creative Commons Attribution 4.0 International license.

### Temperature sensitivity assay.

Strains were grown overnight in YPD broth at 30°C, washed in phosphate-buffered saline (PBS), and set to a concentration of 1 × 10^7^ CFU/ml. Tenfold serial dilutions were performed to reach a final concentration of 1 × 10^2^ CFU/ml, and 4-µl amounts from each dilution were spotted onto YPD plates, incubated at 30°C, 37°C, and 42°C for 2 days, and imaged.

### Liquid hypha formation.

To assess hypha formation in liquid, strains were grown overnight in YPD at 30°C and transferred to YPD containing 10% fetal bovine serum as described in reference 15. Cells were grown for 4 h at 225 rpm at 37°C. Cells were imaged using a Zeiss Axio Imager A1 fluorescence microscope equipped with an AxioCam MRm digital camera.

### Biofilm formation.

Biofilms were grown on contact lenses according to a protocol published previously (43). Briefly, each strain was grown in a shaking incubator at 37°C for 20 h at 225 rpm. Cultures were then centrifuged at 2,500 × *g* for 5 min at 4°C. The supernatant was removed, and the cell pellet was resuspended in fresh PBS. Cells were washed three times, and then the cells were resuspended in PBS to achieve a final concentration of 1 × 10^7^ CFU/ml. Individual contact lenses (Acuvue) were transferred to a 12-well tissue culture plate. Contact lenses were submerged in PBS for 15 min before being transferred to a new 12-well plate. Two contact lenses per strain were used. To each contact lens, 4 ml of the respective *Candida* cell suspension was added and allowed to incubate (without shaking) at 37°C for 90 min to permit cell attachment. Following attachment, each contact lens was carefully transferred to a new 12-well plate containing 4 ml of YNBD (0.67% yeast nitrogen base without amino acids, 2% glucose) medium. Plates containing the lenses were then incubated for 22 h at 37°C with gentle shaking at 50 rpm.

### Scanning electron microscopy.

Samples were fixed with 4% paraformaldehyde (Electron Microscopy Sciences) in PBS for 45 min at room temperature. Samples were rinsed twice with PBS and then dehydrated through a series of ethanol washes (30%, 50%, 70%, 90%, and 100%). The samples were subsequently coated with gold and imaged with a field emission scanning electron microscope (FESEM) (FEI XL30) in high-vacuum mode at 5 kV.

### **Accession number**(**s).**

The coordinates and structure factors of *C. albicans* Tps1 in complex with UDPG, UDP and G6P, and UDP and VDM have been deposited in the Protein Data Bank under accession codes 5HUT, 5HUU, and 5HVL. The coordinates and structure factors of *A. fumigatus* Tps1A and Tps1B bound to UDP and VDM have been deposited in the Protein Data Bank under accession codes 5HVM and 5HVO.
