# Supplementary material for: Structural and In Vivo Studies on Trehalose-6-Phosphate Synthase from Pathogenic Fungi Provide Insights into Its Catalytic Mechanism, Biological Necessity, and Potential for Novel Antifungal Drug Design
Source: mBio. 2017 Jul 25;8(4):e00643-17. doi: 10.1128/mBio.00643-17 (PMC5527307; doi:10.1128/mBio.00643-17)
Supplement: FIG S1 [file mbo004173405sf1.docx]

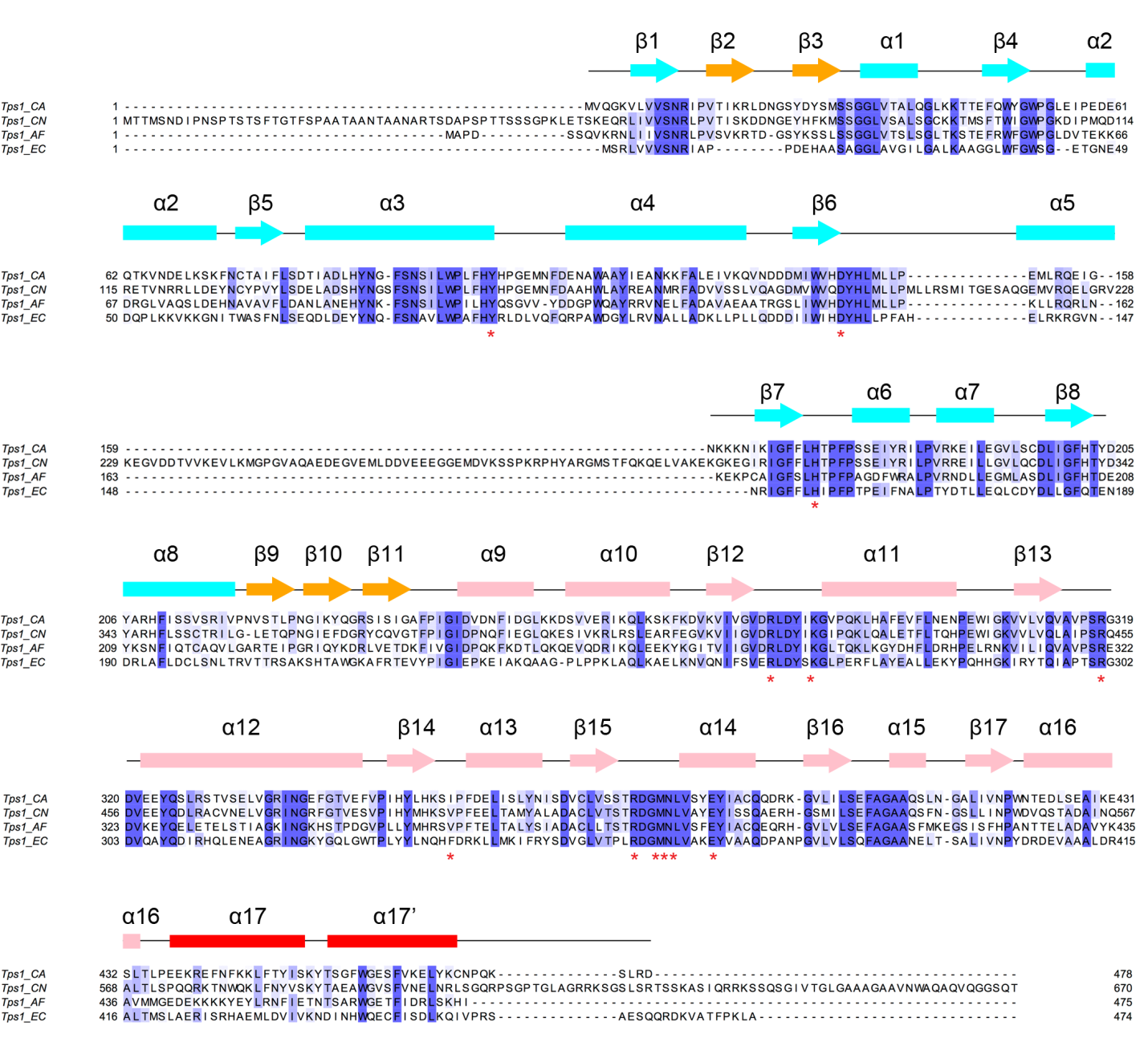


**Figure S1. Structure-based sequence alignment of *C. albicans* Tps1 with Tps1 proteins from other species.**

Tps1 sequences of *C. albicans* (Q92410)*, Cryptococcus neoformans* (Q6IVK9)*, A. fumigatus (*A0A084BNL5-Tps1B) *and E. coli* (P31677) are aligned. The sequences are annotated as *Tps1_CA*, *Tps1_CN*, *Tps1_AF* and *Tps1_EC*, respectively. The secondary structure of *C. albicans* Tps1 is shown above the sequences whereby α helices are depicted by arrowheads and β strands by rectangles. Identical residues found in all four Tps1 proteins are contained in blue boxes whilst those identical in 3 of 4 proteins are contained in light blue boxes. The secondary structure elements are colored as in Figure 2. Key substrate binding residues are highlighted by red asterisks.
