# Supplementary material for: Structural and In Vivo Studies on Trehalose-6-Phosphate Synthase from Pathogenic Fungi Provide Insights into Its Catalytic Mechanism, Biological Necessity, and Potential for Novel Antifungal Drug Design
Source: mBio. 2017 Jul 25;8(4):e00643-17. doi: 10.1128/mBio.00643-17 (PMC5527307; doi:10.1128/mBio.00643-17)
Supplement: TABLE S1 [file mbo004173405st1.docx]

| **Table A1. Selected *C. albicans* Tsp1 Data Collection and Refinement Statistics**^a^ | | | |
| --- | --- | --- | --- |
|  | *C. albicans* Tps1- UDPG | *C. albicans* Tps1 - (UDP+G6P) | *C. albicans* Tps1 - (UDP+VDM) |
| **Data Collection** |  |  |  |
| Space group | P6_4_ | P6_4_ | P6_5_22 |
| Unit cell  a, b, c (Å)  α, β, γ (°) | 98.7, 98.7, 187.7  90, 90,120 | 98.7, 98.7, 188.9  90, 90,120 | 115.6, 115.6, 282.7  90, 90,120 |
| Resolution (Å) | 50.0-1.90 (1.93-1.90) | 50.0-2.37 (2.41-2.37) | 50.0-1.80 (1.83-1.80) |
| Wavelength (Å) | 1.000 | 1.000 | 1.000 |
| Completeness (%) | 99.9 (99.9) | 99.6 (97.3) | 99.6 (100) |
| Redundancy | 5.8 (5.6) | 5.7 (5.6) | 10.3 (10.7) |
| I/σI | 21.5 (2.5) | 18.5 (2.0) | 51.6 (4.9) |
| R_merge_ ^b^ | 0.07 (0.672) | 0.075 (0.638) | 0.092 (0.666) |
| **Refinement** |  |  |  |
| R_work_ ^c^ [%] | 17.9 | 20.0 | 17.9 |
| R_free_ ^d^ [%] | 20.0 | 24.7 | 20.6 |
| RMS deviations |  |  |  |
| Bonds [Å] | 0.008 | 0.003 | 0.007 |
| Angles [^o^] | 1.18 | 0.679 | 1.113 |
| B factors (Å^2^) |  |  |  |
| Overall | 35.7 | 62.5 | 32.0 |
| Proteins | 35.4 | 62.6 | 31.2 |
| Ligand/ion | 35.8 | 57.0 | 28.3 |
| Water | 40.5 | 54.2 | 40.0 |
| Ramachandran [%] |  |  |  |
| Favored, outlier | 97.4, 0.3 | 95.9, 0.4 | 97.2, 0.2 |
| ^a^ statistics for the highest resolution shell are shown in parentheses.  ^b^ R_merge_ = Σ\|I- 〈I〉\|/ Σ\|I\|, where I is the observed intensity and <I> is the average intensity of several symmetry-related observations.  ^c^ R_work_ = Σ\|\|F_o_\|-\|F_c_\|\|/ Σ\|F_o_, where F_o_ and F_c_ are the observed and calculated structure factors, respectively.  ^d^ R_free_ = Σ\|\|F_o_\|-\|F_c_\|\|/ Σ\|F_o_ for 5% of the data not used at any stage of the structural refinement. | | | |
