# Supplementary material for: Structural and In Vivo Studies on Trehalose-6-Phosphate Synthase from Pathogenic Fungi Provide Insights into Its Catalytic Mechanism, Biological Necessity, and Potential for Novel Antifungal Drug Design
Source: mBio. 2017 Jul 25;8(4):e00643-17. doi: 10.1128/mBio.00643-17 (PMC5527307; doi:10.1128/mBio.00643-17)
Supplement: FIG S2 [file mbo004173405sf2.docx]

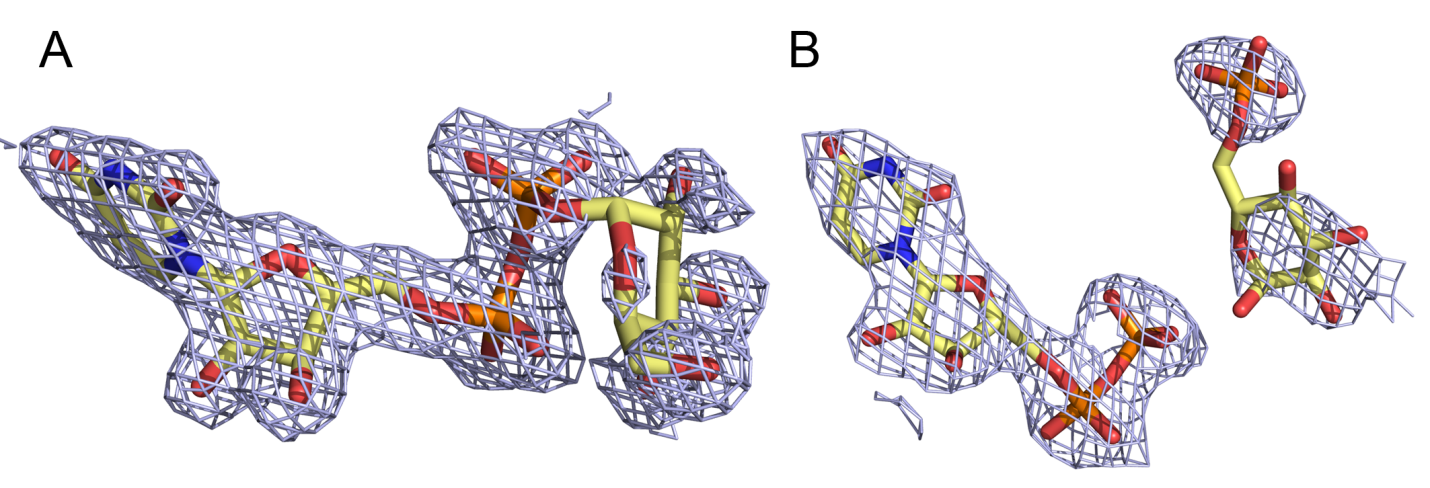


**Figure S2. Electron density of the UDPG and (UDP + G6P) substrates/product in the structure of the Tps1-UDPG and Tps1-(UDP + G6P) complexes.**

(A,B) 2F_o_-F_c_ electron density maps of the bound substrates. The electron density is shown as a light blue mesh and contoured at 1.5 σ. Substrates are shown as atom-colored yellow sticks.
