# Supplementary material for: Structural and In Vivo Studies on Trehalose-6-Phosphate Synthase from Pathogenic Fungi Provide Insights into Its Catalytic Mechanism, Biological Necessity, and Potential for Novel Antifungal Drug Design
Source: mBio. 2017 Jul 25;8(4):e00643-17. doi: 10.1128/mBio.00643-17 (PMC5527307; doi:10.1128/mBio.00643-17)
Supplement: FIG S3 [file mbo004173405sf3.docx]

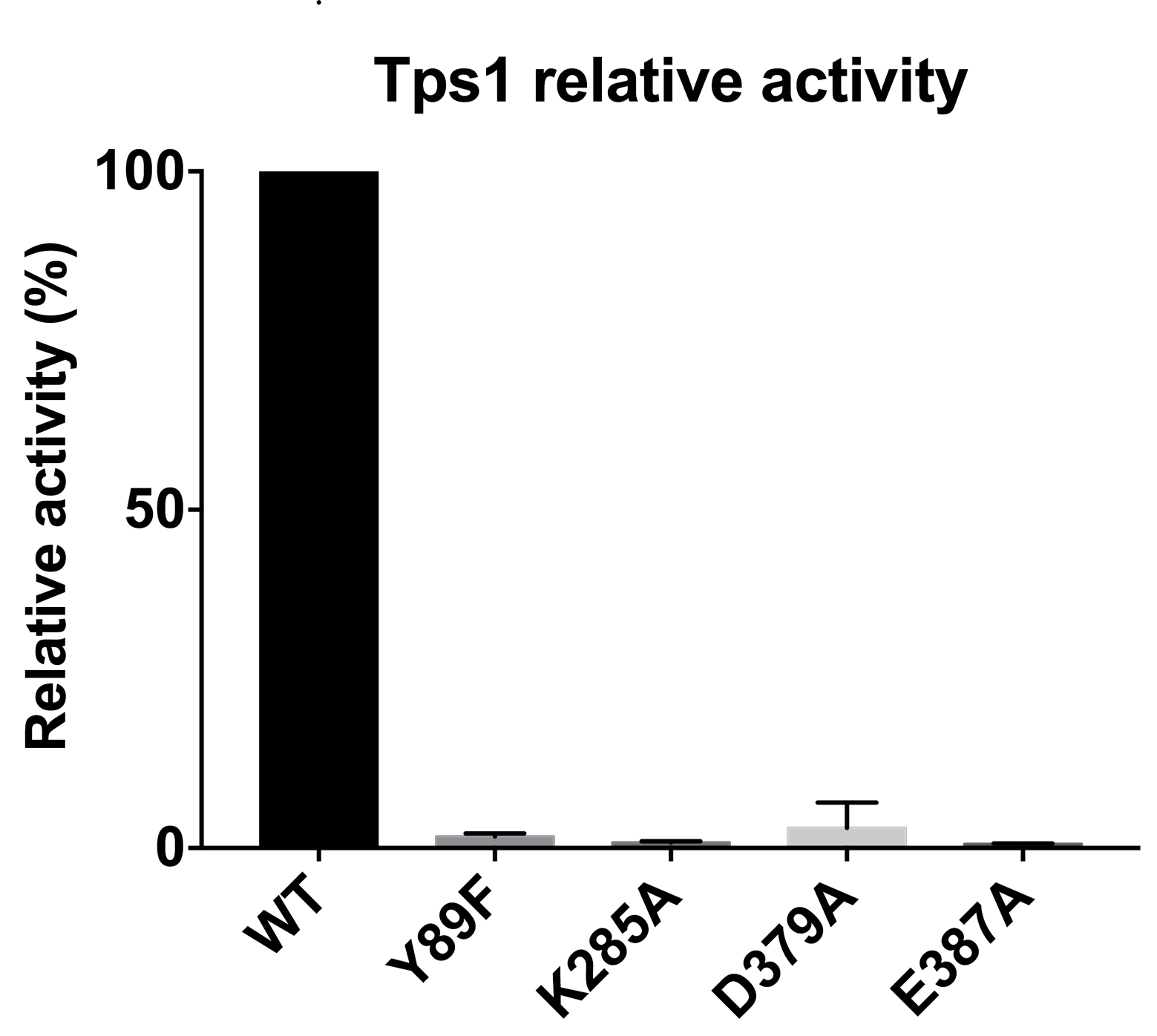


**Figure S3. Relative catalytic activity of *C. albicans* WT Tps1 and selected mutants.**

Error bars represent S.E. of three independent measurements.
