# Supplementary material for: Structural and In Vivo Studies on Trehalose-6-Phosphate Synthase from Pathogenic Fungi Provide Insights into Its Catalytic Mechanism, Biological Necessity, and Potential for Novel Antifungal Drug Design
Source: mBio. 2017 Jul 25;8(4):e00643-17. doi: 10.1128/mBio.00643-17 (PMC5527307; doi:10.1128/mBio.00643-17)
Supplement: FIG S4 [file mbo004173405sf4.docx]

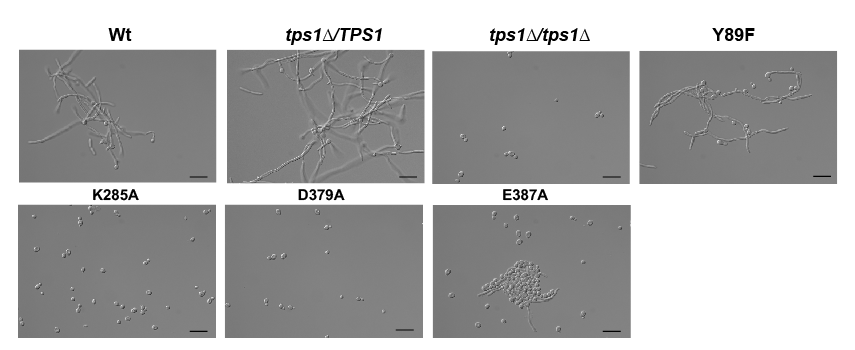


**Figure S4. Effect of *C. albicans* Tps1 point mutations on formation of hyphae in the presence of serum.**

A *tps1∆/tps1∆* is defective in its ability to form hyphae as previously reported. Strains carrying point mutations, K285A, D379A, and E387A, also failed to form hyphae. Yeast cells are present for Y89F suggesting that this mutation does impact hyphal formation but to a lesser degree compared to the other single point mutations. Overnight cultures were washed and transferred to YPD containing 10% fetal bovine serum and incubated at 37 ˚C for 4 h with shaking. The scale bars represent 20 microns.
