# Supplementary material for: Structural and In Vivo Studies on Trehalose-6-Phosphate Synthase from Pathogenic Fungi Provide Insights into Its Catalytic Mechanism, Biological Necessity, and Potential for Novel Antifungal Drug Design
Source: mBio. 2017 Jul 25;8(4):e00643-17. doi: 10.1128/mBio.00643-17 (PMC5527307; doi:10.1128/mBio.00643-17)
Supplement: TABLE S2 [file mbo004173405st2.docx]

| **Table A2. Selected *A. fumigatus* Tps1A and Tps1B Data Collection and Refinement Statistics**^a^ | | | |  |
| --- | --- | --- | --- | --- |
|  | *A. fumigatus* Tps1A - (UDP+VDM) | *A. fumigatus* Tps1B - (UDP+VDM) | |  |
| **Data Collection** |  |  | |  |
| Space group | P2_1_22_1_ | P22_1_2_1_ | |  |
| Unit cell  a, b, c (Å)  α, β, γ (°) | 83.9, 101.2, 147.0  90, 90, 90 | 88.3, 130.6, 184.8  90, 90,90 | |  |
| Resolution (Å) | 50.0-2.80 (2.85-2.80) | 50.0-2.46 (2.50-2.46) | |  |
| Wavelength (Å) | 1.000 | 1.000 | |  |
| Completeness (%) | 99.9 (100.0) | 97.6 (98.9) | |  |
| Redundancy | 4.8 (4.5) | 5.0 (4.3) | |  |
| I/σI | 16.3 (2.1) | 17.3 (2.2) | |  |
| R_merge_ ^b^ | 0.103 (0.882) | 0.107 (0.682) | |  |
| **Refinement** |  |  | |  |
| R_work_ ^c^ [%] | 17.6 | 20.0 | |  |
| R_free_ ^d^ [%] | 23.1 | 23.6 | |  |
| RMS deviations |  |  | |  |
| Bonds [Å] | 0.008 | 0.008 | |  |
| Angles [^o^] | 1.21 | 1.21 | |  |
| B factors (Å^2^) |  |  | |  |
| Overall | 66.3 | 51.8 | |  |
| Protein | 66.4 | 51.6 | |  |
| Ligand/ion | 58.3 | 44.9 | |  |
| Water | 55.1 | 48.6 | |  |
| Ramachandran [%] |  |  | |  |
| Favored, outlier | 98.0, 0.3 | 97.0, 0.5 | |  |
| ^a^ statistics for the highest resolution shell are shown in parentheses.  ^b^ R_merge_ = Σ\|I- 〈I〉\|/ Σ\|I\|, where I is the observed intensity and <I> is the average intensity of several symmetry-related observations.  ^c^ R_work_ = Σ\|\|F_o_\|-\|F_c_\|\|/ Σ\|F_o_, where F_o_ and F_c_ are the observed and calculated structure factors, respectively.  ^d^ R_free_ = Σ\|\|F_o_\|-\|F_c_\|\|/ Σ\|F_o_ for 5% of the data not used at any stage of the structural refinement. | | | | |
