# Supplementary material for: Structural and In Vivo Studies on Trehalose-6-Phosphate Synthase from Pathogenic Fungi Provide Insights into Its Catalytic Mechanism, Biological Necessity, and Potential for Novel Antifungal Drug Design
Source: mBio. 2017 Jul 25;8(4):e00643-17. doi: 10.1128/mBio.00643-17 (PMC5527307; doi:10.1128/mBio.00643-17)
Supplement: FIG S5 [file mbo004173405sf5.docx]

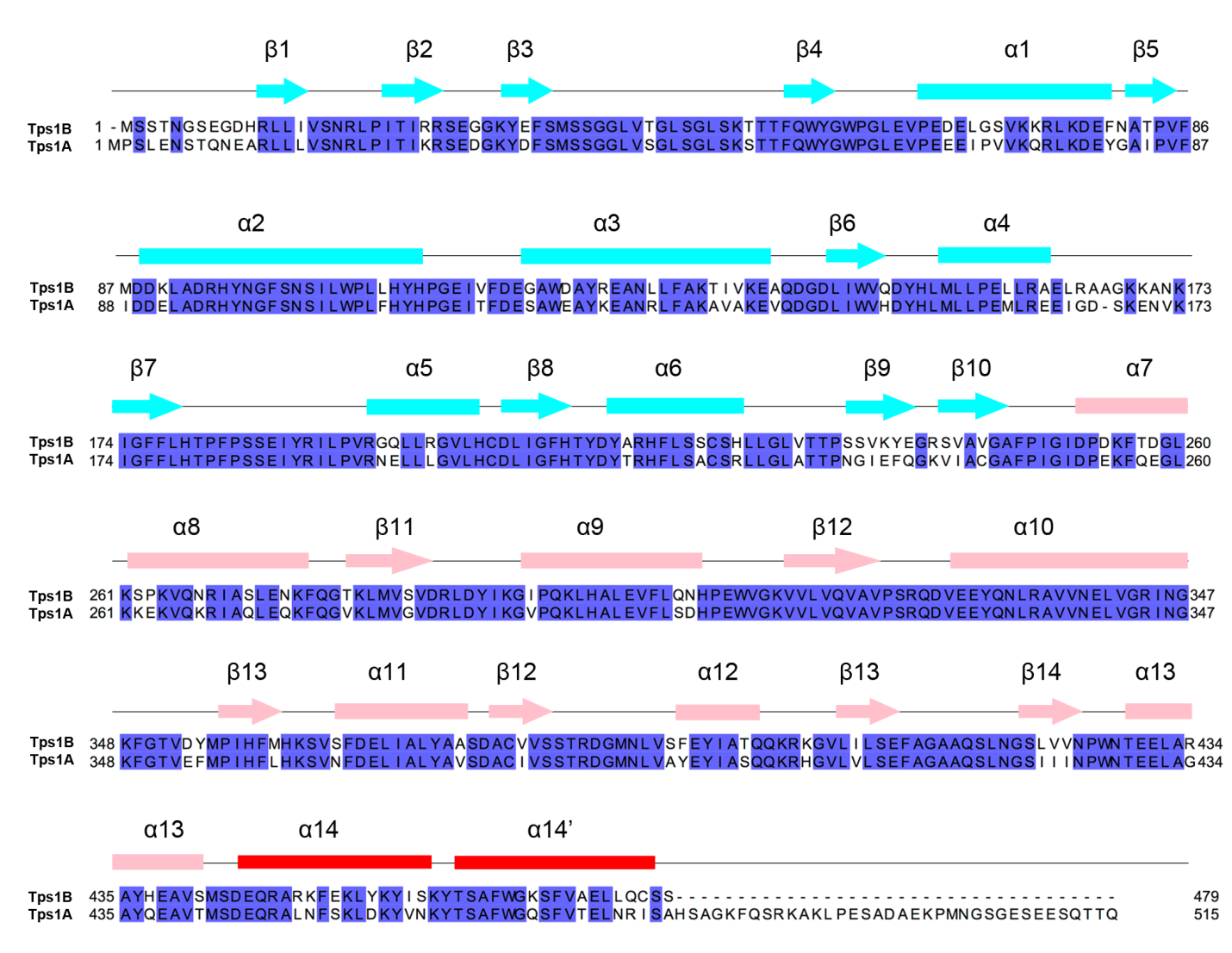


**Figure S5. Sequence alignment of *A. fumigatus* Tps1A and Tps1B.**

Primary sequences of *A. fumigatus* Tps1B and Tps1A are aligned. Identical sequences are shaded blue. The secondary structure of Tps1B is shown above the alignment. Arrows represent α helices and rectangles β strands. The secondary structure elements are colored as in Fig. 5. Note the C-terminal extension of Tps1A.
