# Supplementary material for: Structural and In Vivo Studies on Trehalose-6-Phosphate Synthase from Pathogenic Fungi Provide Insights into Its Catalytic Mechanism, Biological Necessity, and Potential for Novel Antifungal Drug Design
Source: mBio. 2017 Jul 25;8(4):e00643-17. doi: 10.1128/mBio.00643-17 (PMC5527307; doi:10.1128/mBio.00643-17)
Supplement: TABLE S3 [file mbo004173405st3.docx]

| **Table A3. Strains used in this study** | | | |  |  |  |  |  |  |
| --- | --- | --- | --- | --- | --- | --- | --- | --- | --- |
| Strains |  | Genotype | Parental strain |  |  |  |  |  | Reference |
| SC5314 |  |  |  |  |  |  |  |  | [43] |
| *tps1∆/TPS1* |  | *tps1∆::loxP/TPS1* | SC5314 |  |  |  |  |  | This study |
| *tps1∆/tps1∆* |  | *tps1∆::loxP/tps1∆::loxP* | *tps1∆/TPS1* |  |  |  |  |  | This study |
| Strains with point mutations | Strain alias |  |  | AA | Mutation | Position | Wt codon | Mt codon |  |
| Y89F | 1pm1 | *tps1∆::loxP/TPS1::TPS1^Y89F^-loxP* | *tps1∆/TPS1* | Y | F | 89 | UAU | UUU | This study |
| K285A | 1pm2 | *tps1∆::loxP/TPS1::TPS1^K285A^-loxP* | *tps1∆/TPS1* | K | A | 285 | AAA | GCU | This study |
| D379A | 1pm3 | *tps1∆::loxP/TPS1::TPS1^D379A^-loxP* | *tps1∆/TPS1* | D | A | 379 | GAU | GCU | This study |
| E387A | 1pm4 | *tps1∆::loxP/TPS1::TPS1^E387A^-loxP* | *tps1∆/TPS1* | E | A | 387 | GAG | GCU | This study |
